# Effect of pore air escape and cement dissolution on granite residual soil disintegration, southeast China

**DOI:** 10.1038/s41598-025-92578-5

**Published:** 2025-03-26

**Authors:** Liping Liao, Dongming Yi, Yinghui Tan, Shaokun Ma, Yunchuan Yang, Zhengwei Liu

**Affiliations:** 1 College of Civil Engineering and Architecture, Guangxi University, Nanning, 530004 China; 2 Key Laboratory of Disaster Prevention and Structural Safety of Ministry of Education, Guangxi University, Nanning, 530004 China; 3 Guangxi Key Laboratory of Disaster Prevention and Engineering Safety, Guangxi University, Nanning, 530004 China; 4 State Key Laboratory of Featured Metal Materials and Life-Cycle Safety for Composite Structure, Guangxi University, Nanning, 530004 China

**Keywords:** Granite residual soil, Pore air escape, Cement dissolution, Disintegration mechanism, Natural hazards, Civil engineering

## Abstract

Granite residual soil in south and southeast China has obvious characteristic of disintegration, which induces collapse erosion and shallow landslide. Although the escape of pore air can be observed in the present hydrostatic water tests, and pore air and cementitious substance dissolution is qualitatively confirmed to promote soil disintegration, these two factors fail to be monitored in real time throughout the whole disintegration process. Therefore, this study utilized two self-developed instruments to monitor the volume of escaping pore air and the process of soil disintegration, and observe the current variations in the solution during the soil disintegration. The disintegration patterns and rates of residual soils with various compaction levels and initial moisture contents were analyzed. The intrinsic affecting mechanisms of pore air escape and cementitious substance dissolution on the soil disintegration were revealed. The results consist of three aspects. (1) The initial moisture content has a significant impact on disintegration pattern, completion time and velocity. (2) The variance in the effect of the increase in compaction degree on the completion time and speed of disintegration is governed by the initial moisture content. (3) The escape of pore air and the dissolution of cementitious substances constitute two principal phenomena during the entire disintegration process of granite residual soil. (4) The repulsive stress generated by the compressed and escaping pore air being greater than the sum of suction stress and viscous stress generated by capillary water and cementitious substance is one of the primary causes for granite residual soil disintegration.

## Introduction

Granite residual soil (GRS) is the in-situ weathered residual accumulation of parent rock^1^ and is widely distributed in Malaysia^2^, Brazil^3^, Korea^4,5^, Singapore^6,7^, and China^8–11^. The South and southeast China, especially Guangxi, Fujian, and Guangdong Province, is covered by a large number of GRS^9,10^. GRS is the regional special soil due to different particle gradation and mineral composition^12,13^. However, GRS has similar water-sensitive property^14^ that the soil tends to soften and disintegrate when submersed in water^15,16^. GRS disintegration destroys the structure integrity of soil and causes serious erosion^10,17^, geological hazard including collapse^18,19^ and shallow landslide^20,21^. These hazards not only cause huge loss of life and property, but also seriously damage highway and local farmland. Therefore, studying the disintegration characteristics and mechanism of GRS has the important scientific significance for preventing and controlling geo-hazard in GRS region.

Although the study on GRS property originates in the 1960s^22^, extensive attention on GRS disintegration begins in the 21th century. A large number of laboratory tests focus on the impact factors, characteristics and mechanism of GRS disintegration. Soil engineering properties are the main factors, including dry density^23^, initial moisture content^17,24,25^, pH^26^, weathering degree^27^. Especially, the effect of initial dry density and water content on GRS disintegration is significantly heterogeneous. Disintegration rate of some GRSs decreases as initial water content increases^14^. However, other GRS shows an opposite trend^28^. Moreover, the different disintegration stages of the same GRS indicate distinct moisture-content dependent pattern. The lower content results in the better water stability and the slower disintegration rate in the second stage^25^. This phenomenon is related to the difference of particle content^29^ and the formation of internal pore structure^30,31^. Rainfall^32^ and groundwater level^15^ are two important environmental factors. The wetting and drying cycle of GRS attributes to the changes in rainfall, evaporation and groundwater level, which destroys the cementation and weakens the binding force between soil particles^33^, and accelerates the disintegration rate^26^. The application of external material such as cement and lime can improve disintegration resistance of GRS^12^. In fact, the aforementioned results are governed by variations in the soil internal composition, such as pore structure and cementation properties. The variation of pore structure can induce the pore air to escape or compress. The disintegration process of GRS is accompanied by this phenomenon. Air escaping from the connected pores can disturb the stability of soil structure^34–37^. Semi-connected and closed pores compressed by infiltrated water will destroy the soil micro structure^33,38^. The above researches focus on the qualitative analysis^34,35^. Escaping pore air could lead to a decline in buoyancy, and result in the readings to experience the first increase and the subsequent decrease^39^. The accuracy of disintegration mass and rate are significantly affected. There are two quantitative methods for the effect of pore air escape on GRS disintegration in Guangdong, China. The release of pore air is considered to be uniform^36^. Therefore, this method ignores the actual process of pore air release, and is not suitable for rapid disintegration of soil mass. The other method assumes that the release of pore air is uneven. The buoyancy ratio obtained by parallel experiments is utilized to correct the disintegration rate^14,24^. However, the error of parallel test and disintegration test is large due to the different test conditions. In summary, it is necessary to obtain the pore air escape during the entire disintegration process in real-time and adjust the disintegration rate according to the actual escaping air. In addition, the cementitious substance in the soil serves as the crucial links between soil particles, and directly determines the mechanical properties^40,41^. Some studies have explained the soil disintegration in terms of cementitious substance dissolution, and point out that cementing substance dissolution can change soil property and favors soil disintegration^37,42^. However, quantitative data such as the dissolution rate of cementing substance during the process of soil disintegration is lacking.

Early disintegration tests were conducted in glass cylinders. However, this method has major drawbacks in terms of accurate readings. Many scholars have improved the disintegration device and utilized balances or tension meters instead of float^26,43,44^. This improvement can yield more accurate readings. Nevertheless, this approach requires manual reading of the data at regular intervals. Some scholars employed tension sensors to overcome the aforementioned deficiencies^33,45,46^. They utilized a computer to control the collection time of the tension sensor to obtain high-precision data. The method improved by the above scholars can obtain more accurate readings of the soil disintegration mass. However, the current device cannot monitor the amount of escaping pore air and cementation substance dissolving during the soil disintegration process.

Based on the above background, this study developed two testing instruments to surmount the above defects. One is utilized to monitor the soil disintegration and the volume of escaping pore air in real-time. The other is employed to observe the variation of cementation substance dissolving. The disintegration morphology and velocities of GRS with diverse compaction levels and initial moisture contents were analyzed. The intrinsic affecting mechanism of escaping pore air and cementitious substance dissolution on soil disintegration was revealed.

## Test materials and methods

### Test soil

**Fig. 1:**
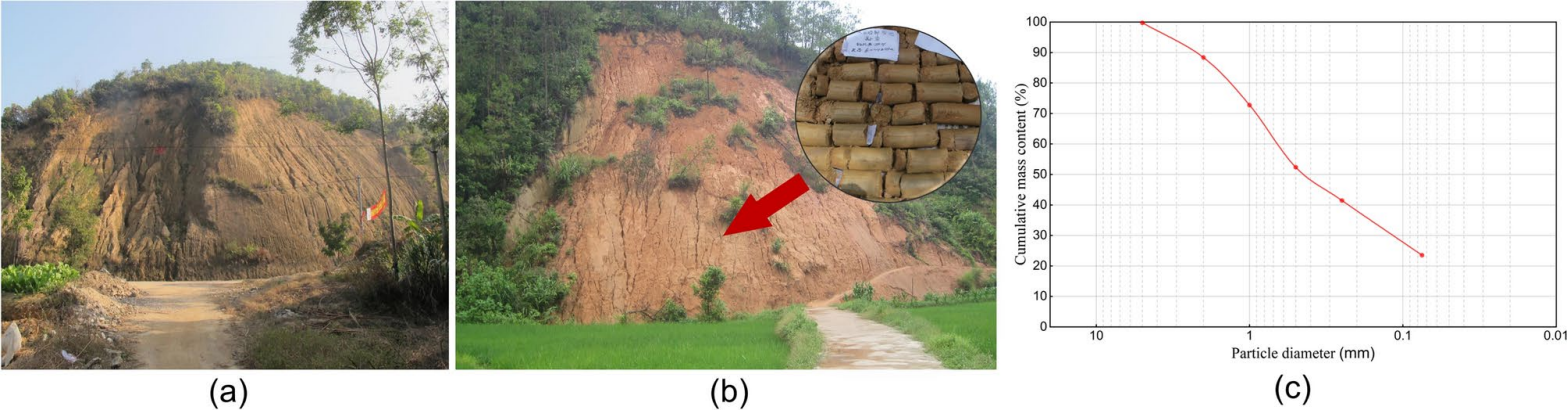
Study area (**a**) Erosion collapse; (**b**) Landslide and soil sample; (**c**) Particle size gradation of GRS.

The test soil was derived from Rong County (E110° 15′~110° 53′, N22° 27′~23° 07′), Yulin City in Guangxi Province of China, where the frequent soil disintegration induces erosion collapse and landslides. The sample depth ranges from 1.5 to 2 m. This study conducts the basic mechanical tests and screening tests based on the Chinese National Standard—Test Methods for Geotechnical Laboratory (GB/T 50123-2019). The maximum and minimum dry density of the soil is 1.73 g/cm^3^ and 1.18 g/cm^3^, respectively. The optimum moisture content is 17.2%, and the specific gravity of the soil particles is 2.71. Figure 1 indicates that GRS is mainly composed of fine gravel (5 mm ≥ *d* > 2 mm), sand (2 mm ≥ *d* > 0.075 mm), and fine particles (*d* ≤ 0.075 mm). The coarse sand (2 mm ≥ *d* > 0.5 mm) has the highest content of 35.97 %. The contents of medium sand (0.5 mm ≥ *d* > 0.25 mm) and fine sand (0.25 mm ≥ *d* > 0.075 mm) are 10.97% and 17.88%, respectively. The non-uniformity coefficient *C*_u_ and curvature radius *C*_c_ are 17.81 and 1.13, respectively.

### Disintegration test

#### Test methods

Based on the data in Section "Test soil", the experimental scheme is designed and shown in Table 1. The compaction degree (*λ*) is set at 80%, 85%, and 90%. Five levels of initial moisture content (*ω*) are established for each compaction degree. They are 5%, 10%, 15%, 20%, and 25%, respectively.

**Table 1:** Disintegration test scheme.

Compaction degree *λ* (%)	Initial moisture content *ω* (%)
80	5, 10, 15, 20, 25
85	
90	

This paper applied the Chinese National Standard—Test Methods for Geotechnical Laboratory (GB/T 50123-2019) to prepare samples. The bottom diameter and height of ring cutter are 0.0618 m and 0.04 m, respectively. The initial moisture content was ascertained by means of the drying method prior to sample preparation. The amount of water required was calculated based on the initial and designed moisture content. The water was sprayed onto the soil by a sprayer. When the soil and water were thoroughly mixed, they were placed in a glass container and sealed for 24 h. Subsequently, the soil moisture content was measured. The maximum allowable difference between the actual value and the target value was  ± 1%. When the soil moisture content fulfilled the requirements, the mass of wet soil was calculated based on compaction degree, initial moisture content, and ring cutter volume. Finally, the wet soil was poured into the ring cutter and pressed into a ring sample.

#### Test instruments

**Fig. 2:**
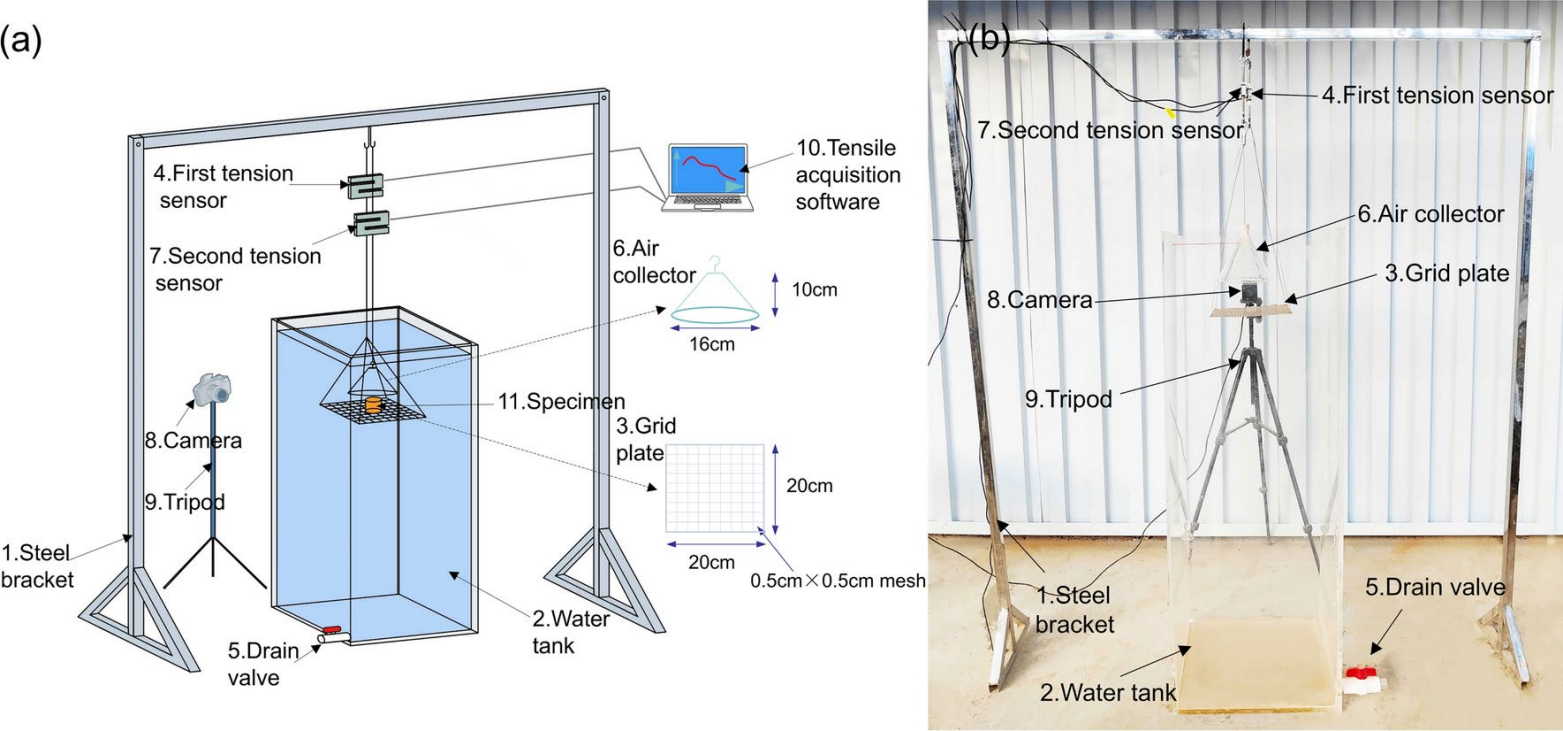
Diagram of disintegration test apparatus (**a**) Schematic diagram; (**b**) Physical diagram.

A self-developed disintegration testing instrument consists of three parts: disintegration device, air collection device and data-acquisition system (Fig. 2). The disintegration device includes a steel bracket (1), a water tank (2), and a grid plate (3) with a first tension sensor (4). The steel bracket with the height of 1.5 m is placed firmly on a flat surface for hanging the tension transducer. The water tank is customized from acrylic material with a length, width and height of 0.4 m × 0.4 m × 1 m. The water tank is equipped with a drain valve (5). The size of the grid plate is 0.2 m × 0.2 m, and the mesh size is 0.005 m × 0.005 m. The accuracy of the first pull sensor is 0.1 g. Air collection device comprises the air collector (6) with a second tension sensor (7). This collector has a cylindrical vitreous body. When the collector is submerged in water, its weight is greater than the maximum buoyancy created by the trapped air. The accuracy of the second pull sensor is 0.05 g.

The temperature of the test water was 20 °C and the pH was 7. Before the experiment started, the installation status of the instrument was checked, and the drain valve was closed. Subsequently, water was pumped into the tank. The water level was controlled 0.1 m from the top of water tank. The air collector was filled with water and immersed in water, and was suspended from the second tension sensor via a small hook on the top surface. At this point, the air collector was located directly above the grid plate. The camera (8) was mounted on a tripod (9) and aimed at the grid plate. Subsequently, the data from the two tension sensors were cleared. The camera and the tensile acquisition software (10) were turned on, and the specimen (11) was placed on the grid plate. The camera and software were not turned off until the sample has completely disintegrated. The time when the first pull sensor reading begins to change is the initial moment of disintegration. If the readings of the two tension sensors do not change by more than 1 g within 120 s, the soil disintegration is considered completely.

The operating principle of the air collection apparatus is described as follows. When the escaping air entered the air collector, the same volume of water was discharged. The air exerted an upward buoyancy force on the top surface of the collector, which caused the reading of the second pull sensor to decrease. According to Archimedes’ principle, the Eqs. (1) and (2) are shown as follows.12where *ρg* is the liquid bulk weight with the unit of g/cm^3^; *V* is the volume of air escaping from the sample at time *t* with the unit of cm^3^; *F*_*b*_ is the buoyancy force generated in the air collection device with the unit of N; *F*_2_ is the change in reading of the second pull sensor with the unit of g.

When the Eq. (2) is substituted into the Eq. (1), the volume of the escaping air shown in Eq. (3) is obtained.3

#### Disintegration index

##### Disintegration rate *R*(*t*)

Disintegration rate *R*(*t*) is the percentage of the disintegration mass at a certain moment *t* to the initial mass. The sample was subjected to mechanical equilibrium analysis, and the Eqs. (4) and (5) were obtained:45where *F*_1_(0) is the reading of the first pull sensor when the sample is first placed on the grid plate, in g; *M* is the initial mass, in g; *V*_0_ is the initial total volume of the sample, in cm^3^; *F*_1_(*t*) is the reading of the first pull sensor at a given moment *t*, in g; *f*(*t*) is the buoyancy of the un-disintegrated soil in water at a certain time *t*, in N; *ρ* is the density of water, and is equal to 1 g/cm^3^; *g* is the acceleration of gravity, and taken as 9.8 m/s^2^.

The pores and mass inside the sample are assumed to be uniformly distributed. Base on the Archimedes’ law, Eq. (6) was obtained:6where *V*_a_(t) is the volume of air escaping from the pore due to water absorption by the sample at a certain moment* t*, in cm^3^; *V*_a_ is the total volume of air inside the soil, in cm^3^.

Equations (6) and (4) were substituted into Eq. (5) to obtain the Eq. (7):7

The second pull sensor was analyzed by the force, and the following equations were obtained:8910where *F*_2_(*t*) is the second pull sensor change reading at a certain moment* t*, in g; *f*_a_(*t*) is the buoyancy force exerted on the air collector by the escaping air at a certain moment *t*, in N; *F*_2_(*n*) is the second pull sensor change reading at the completion of disintegration, in g.

Equations (8), (9) and (10) were substituted into Eq. (7) to obtain the Eq. (11):11

Equation (11) is translated to the disintegration rate *R*(*t*) as follows:12

##### Disintegration velocity *v*_t_

*V*_t_ is the velocity of the disintegration amount over disintegration time in a certain period of time, and it is calculated by Eq. (13):13

##### Air escaping rate *R*_a_(*t*)

*R*_a_(*t*) is the ratio of the amount of air escaping from the soil at time* t* to the total amount of air escaping when the disintegration is completed. Since some soil samples cannot be completely disintegrated, it is approximately assumed that the air escape rate is equal to the soil sample disintegration rate when the disintegration is completed. *R*_a_(*t*) is calculated by Eq. (14):14

where *R*(*n*) is the disintegration rate when the soil disintegration is complete.

### Ion micro-current test

#### Test methods

**Fig. 3:**
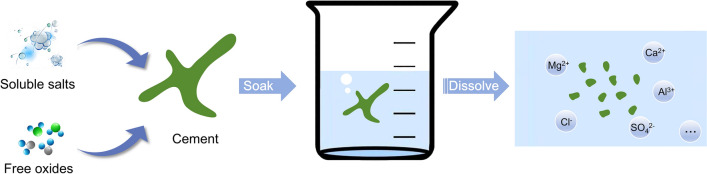
Generation and dissolution of cement.

**Fig. 4:**
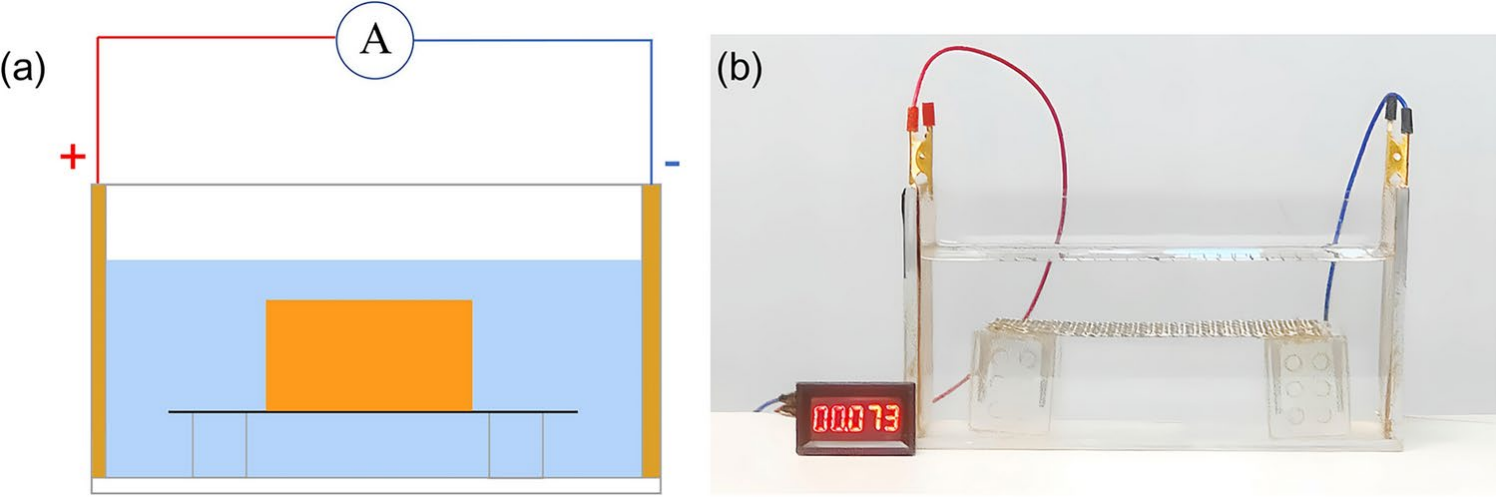
Solution current measurement device: (**a**) Schematic diagram; (**b**) Physical diagram.

The cementitious substance of GRS composed of soluble salts and free oxides can generate Ca^2^^+^, Mg^2^^+^, Cl^-^ and other ions when it dissolves in water^47,48^ (Fig. 3). The conductivity of a static aqueous solution depends on the temperature and ion concentration of the solution. During the disintegration of the sample, the dissolution of cementitious substance increases the ionic concentration of the solution. The conductivity of the solution can be enhanced accordingly. A self-developed apparatus consisting of a small disintegration tank and an ammeter was employed to measure the change of solution current during the disintegration of the sample. The ion concentration could be reflected by analyzing the solution current. The both sides of disintegration tank were affixed by the conductive copper sheets (Fig. 4). The model of the ammeter is KV-AMP070mA, which has a measurement accuracy of 1 µA and a maximum range of 7 × 10^5^ µA. The ammeter can measure the change of current in real time.

In order to eliminate the effect of solution ionization on the test, high purity deionized distilled water were adopted as the disintegration liquid. The samples in this test have the same compaction degree and initial moisture content as the sample mentioned in Section "Disintegration test". A camera was applied to record the changes in the current meter readings during the experiment. The difference between the disintegration completion time in this section and the disintegration completion time in Section "Disintegration test" is required to be no more than 5%.

#### Dissolution rate of cementitious substance

The magnitude of the current measured by this device is associated with the temperature of the solution, the concentration of ions, and the change of water level. The temperature of the disintegrating solution is constant. The dissolution of cementitious substance results in an increase in ion concentration and current. However, the escaping pore air could cause the water level of the solution to drop slightly. This decreases the measured current. In this test, the total amount of current increase at the completion of disintegration is measured to be 600~1900 μA. The maximum current reduction due to air escape is only 3.2 μA. Therefore, this value could be disregarded. The dissolution rate of the cementation substance is calculated by Eq. (15).15where *R*_c_(*t*) is the dissolution rate of cementation substance, in %; *I*(*t*) is the ammeter reading at a certain time *t*, in μA; *I*(0) is the initial reading of the galvanometer when the specimen is completely immersed in water, in μA; *I* is the ammeter reading at the completion of disintegration, in μA.

## Test results and analysis

### Disintegration test results

#### Disintegration pattern

**Fig. 5:**
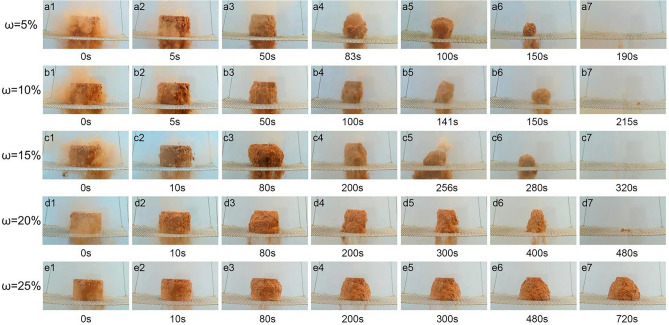
Disintegration process of samples with *λ* of 85%

Because the patterns of the disintegration process with *λ* of 80%, 85%, and 90% are similar, this section mainly describes the disintegration pattern with *λ* of 85%. Figure 5 illustrates that when *ω* ranges from 5 to 15%, the disintegration process can be divided into three stages: water-absorbing disintegration, rapid disintegration, and collapse disintegration. When the specimen is immersed in water, the soil particles of the surface are separated from the specimen, which cause the surrounding water to become turbid. During the first 10 s, the weight of specimen is increased by absorption water and the escape of air bubbles. This stage is the water absorption and disintegration stage (Fig. 5a1–a2,b1–b2,c1–c2). Subsequently, the samples continue to slough off in a flocculent manner. Part of the soil is disturbed by the escaping pore air. The detachment of soil particles from the sample is accelerated. This process is the rapid disintegration stage. The sample morphology gradually changes into the elongated cylinder. The samples with *ω* of 5%, 10%, and 15% collapses at 83 s, 141 s, and 256 s, respectively (Fig. 5a3–a5,b3–b5,c3–c5). Subsequently, the samples continue to disintegrate at a steady rate until the end of disintegration (Fig. 5a6–a7,b6–b7,c6–c7).

The disintegration characteristics with *ω* of 20% and 25% are different from those with *ω* of 5–15%. When the sample with *ω* of 20% is immersed in water, the surrounding water becomes slightly turbid (Fig. 5d1). Subsequently, the sample continues to shed in a fragment form. Although the soil becomes slender, it cannot collapse. The sample always disintegrates at a relatively stable rate (Fig. 5d2–d7). When the sample with *ω* of 25% is immersed in water, a small number of particles disperse on the surface. The water is still clear (Fig. 5e1). Subsequently, the samples continue to slough off in a fragmentary manner, and are accompanied by tiny bubbles escaping. Soil disintegration mainly occurs in the upper edge region of the specimen. The disintegration rate is always very slow. Eventually, the soil cannot completely disintegrate, and its shape becomes a cone (Fig. 5e2–e7).

#### Disintegration completion time and velocity

**Fig. 6:**
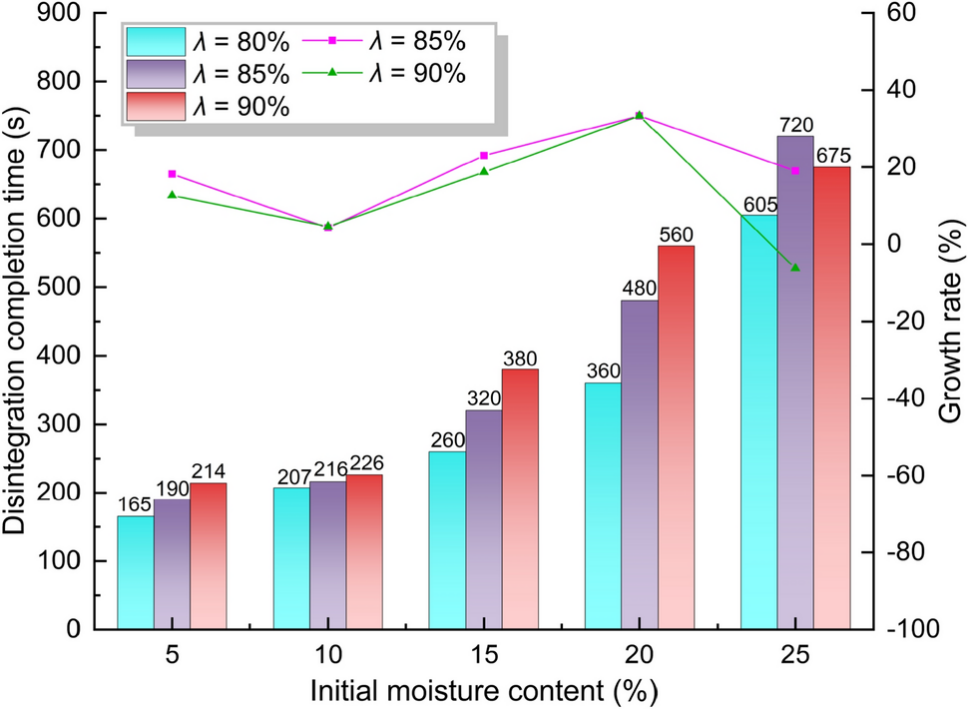
Disintegration completion time and its growth rate.

Figure 6 shows that when *λ* is the same, the disintegration completion time increases with the increase of *ω*. When *λ* is increased by 5% each time, the disintegration completion time with *ω* of 5% and 10% is increased by 25 s and 10 s, respectively. However, when *ω* is 15% and 20%, the disintegration completion time increases as high as 60–120 s as the compaction increases. This indicates that the effect of the increasing compaction degree on the disintegration completion time is related to the initial water content. The utilization of growth rate can visually represent the degree of aforesaid effect. When the initial moisture content remains invariant, the growth rate at *λ* = 85% pertains to the ratio of the difference in disintegration completion time between *λ* = 85% and *λ* = 80% to the time at *λ* = 80%. The growth rate of *λ* = 90% is calculated based on the disintegration completion time between *λ* = 90% and *λ* = 85%. Figure 6 illustrates that when *ω* is 25% and *λ* = 90%, the negative growth rate is ascribed to the circumstance that the disintegration time at 90% is shorter than that at 85%. When *ω* is 10%, *λ* = 85% and *λ* = 90% has the smallest positive growth rate of 4.5%. When *ω* is 20%, *λ* = 85% and *λ* = 90% has the largest positive rate of 33%. This indicates that when *ω* is 10%, the increase in compaction has the least effect on the disintegration completion time. When the *ω* is 20%, the increase in compaction has the greatest effect on the disintegration completion time. When *ω* ranges from 5 to 25%, the growth rate for *λ* = 90% are smaller than that for *λ* = 85%. This indicates that when *λ* is 85%, the continuous increase in *λ* has a little effect on disintegration completion time.

**Fig. 7:**
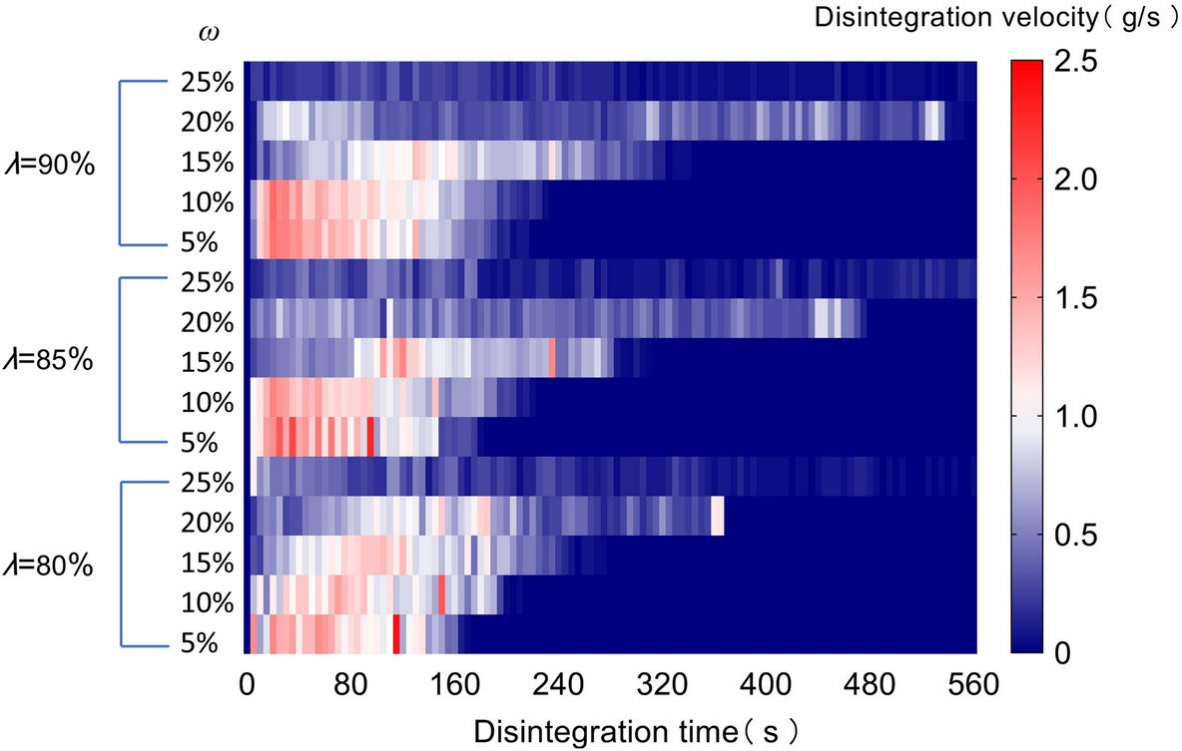
Disintegration velocity of GRS.

The disintegration velocity calculated by Eq. (10) is depicted in Fig. 7. When *λ* = 80%, the disintegration velocity for *ω* of 5% and 10% indicates an initially fast trend followed by a slow tendency. The disintegration velocity for *ω* of 5% is greater than 1.2 g/s during 0–140 s. Subsequently, the disintegration velocity decreases to below 0.5 g/s. The disintegration velocity for *ω* of 10% is greater than 1.0 g/s during 0~160 s. However, the velocity is essentially less than 0.7 g/s after 160 s. The disintegration velocity for *ω* of 5% and 10% instantaneously increases to 2.5 g/s and 2.0 g/s at 120 s and 155 s, respectively. This is because the sample collapse causes a sudden disintegration of a large amount of soil. The disintegration velocity for *ω* of 15% gradually increases from 0 to 1.3 g/s during 0–120 s, and gradually decreases to below 0.5 g/s after 120 s. The disintegration velocity for *ω* of 20% and 15% exhibits similar variation trend. However, the maximum disintegration velocity is only 1.1 g/s. The disintegration velocity for *ω* of 25% is smaller than 0.5 g/s. When *ω* ranges from 5 to 15%, the disintegration velocities of three compaction degrees exhibit the similar trends. When *ω* is 20%, the overall disintegration velocity for *λ* = 85% is basically less than 0.8 g/s. The disintegration velocity for *λ* = 90% reaches 1.0 g/s within the 0–100 s. When *λ* = 85% and *λ* = 90%, the disintegration velocity for *ω* of 25% is very slow, mostly below 0.5 g/s. When *ω* is 25%, the maximum disintegration velocity can decrease as *λ* increases from 80 to 90%.

#### Disintegration rate and air escape rate

**Fig. 8:**
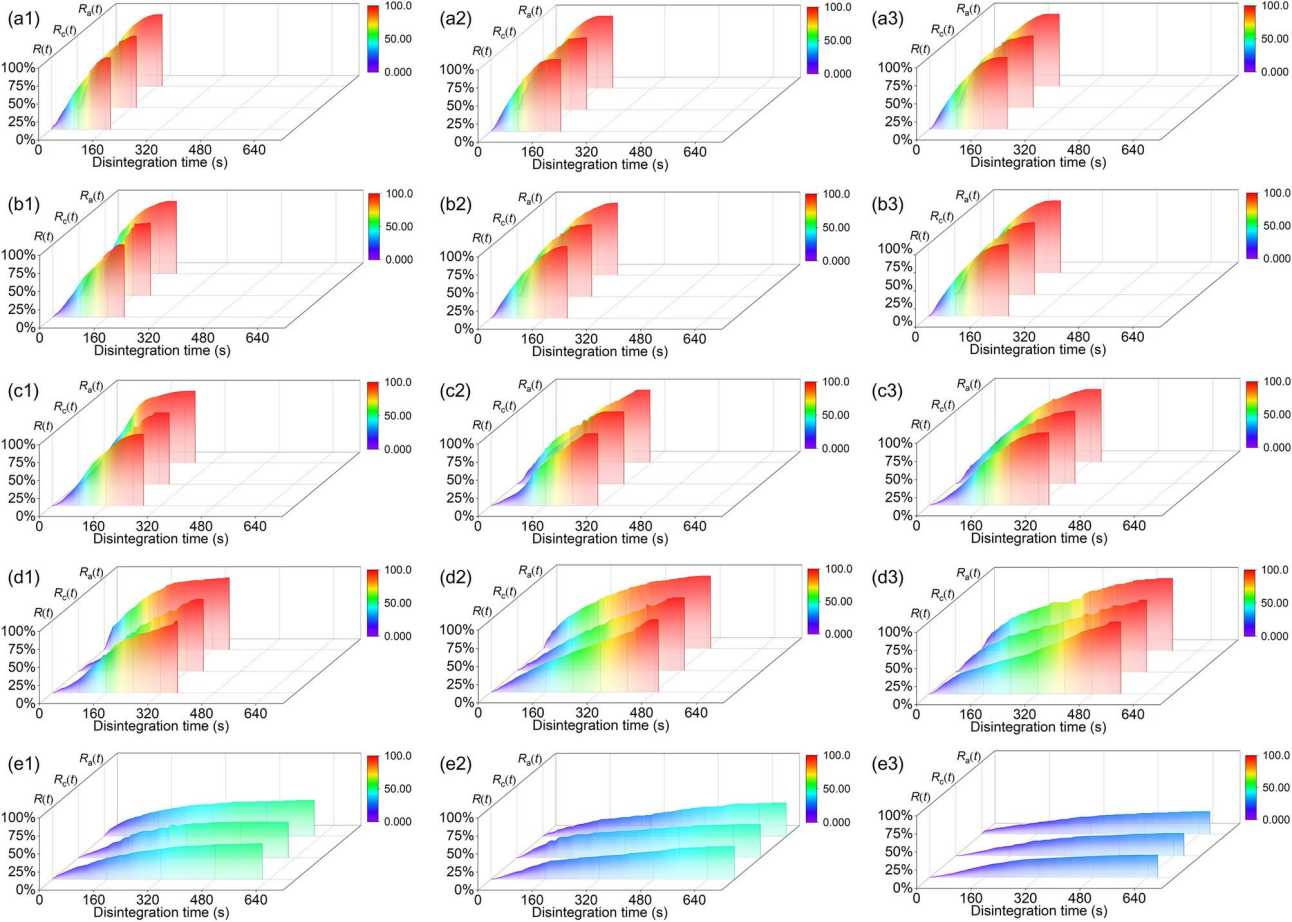
Relationship between disintegration rate* R*(*t*), cementitious substance dissolution rate *R*_c_(*t*), and air escape rate *R*_a_(*t*): (a1–e1) *λ* = 80%; (a2–e2) *λ* = 85%; (a3–e3) *λ* = 90%; a, b, c, d, e represents that *ω* is 5%, 10%, 15%, 20%, and 25%, respectively.

Figure 8a1,b1,c1 illustrates that the disintegration rate curves are relatively similar for *ω* of 5%, 10%, and 15% at *λ* = 80%. The overall slope of each curve is negatively correlated with the initial moisture content. The curve slope with *ω* of 20% is relatively large during 0–180 s (Fig. 8d1). The curve slope for *ω* of 25% gradually decreases, and its shape is similar to a parabola (Fig. 8e1). The final disintegration rate is 49.4%, because the specimen fails to disintegrate completely. The air escape rate within the range of *ω* from 5 to 20% are approximately linear within 0–35 s. The intervals between these four curves are extremely small. This indicates that air rapidly escapes within the initial 35 s. The curve slope of the air escape rate is minimized for *ω* of 25%.

Figure 8a2,b2 indicates that the disintegration rate curves for *ω* of 5% and 10% are extremely similar at *λ* = 85%. These two curves are approximately linear during 0–80 s, and their slopes begin to decrease slowly after 80 s. The disintegration rate for *ω* of 20% is greater than that for *ω* of 15% during 0–95 s (Fig. 8c2,d2). Subsequently, the disintegration rate for *ω* of 15% exceeds the disintegration rate for *ω* of 20%. Figure 5 in Section "Disintegration pattern" shows that the sample with *ω* = 15% undergoes the stage of water absorption and slow disintegration, while this stage is absent when *ω* = 20%. The curves of air escape rate for *ω* of 5% and 10% are extremely similar. Especially in 0–35 s, these two curves almost completely overlap. The air escape rate for *ω* of 20% is greater than that for *ω* of 15% during 0–130s. This relationship changes to the opposite pattern after 130s. The final disintegration rate for *ω* of 25% is 46.1% (Fig. 8e2). The air escape rate is less than the disintegration rate for some time periods. The reason is that during the disintegration process, some tiny air bubbles adsorbing on the soil particles commence to descend and then ascend slowly. This leads to the lag effect of the collection of escaping air. The lag effect is not significant when the volume of escaping bubble is large. The escaping air for *ω* of 25% is dominated by tiny bubbles. This exacerbates the significance of the lag effect, which results in an air escape rate profile that is smaller than the disintegration rate at certain moments.

The disintegration rate curve at *λ* = 90% exhibits some similarities to the curve at *λ* = 85% (Fig. 8a3,b3,e3). The curve for *ω* of 5% and 10% is extremely compact at *λ* is 90% (Fig. 8a3,b3). Especially in 0–40 s, these two curves almost overlap. The disintegration rate for *ω* of 20% is greater than that for *ω* of 15% during 0–100 s (Fig. 8c3,d3). The growth of the disintegration rate for *ω* of 20% becomes slow after 100s. The curve slope of the disintegration rate for *ω* of 25% is extremely small, and a final disintegration rate is 31.2% (Fig. 8e3). The air escape rate curves for *ω* of 5%, 10% almost overlap. This indicates that the process of increasing *ω* from 5 to 10% has the least effect on air escape. When *ω* is 15% and 20%, the air escape rate is basically the same at 0–80 s. The air escape rate for *ω* of 15% is gradually larger than that for *ω* of 20% after 80 s. The air escape rate of *ω* of 20% is larger than that of *ω* of 20%. The escaping air bubbles in the sample with *ω* = 25% is very small, and the lag effect is significant.

When *ω* is 25%, the maximum disintegration rates at *λ * = 80%, 85%, and 90% are 49.4%, 46.1%, and 31.2%, respectively. The maximum disintegration rate decreases as *λ* increases. Figure 6 shows that the disintegration completion time increases as *λ* increases when *ω* ranges from 5 to 20%. This reveals that when *ω* ranges from 5 to 25%, the disintegration resistance of GRS can be improved as *λ* increases from 80 to 90%.

### Ion micro-current test results

**Fig. 9:**
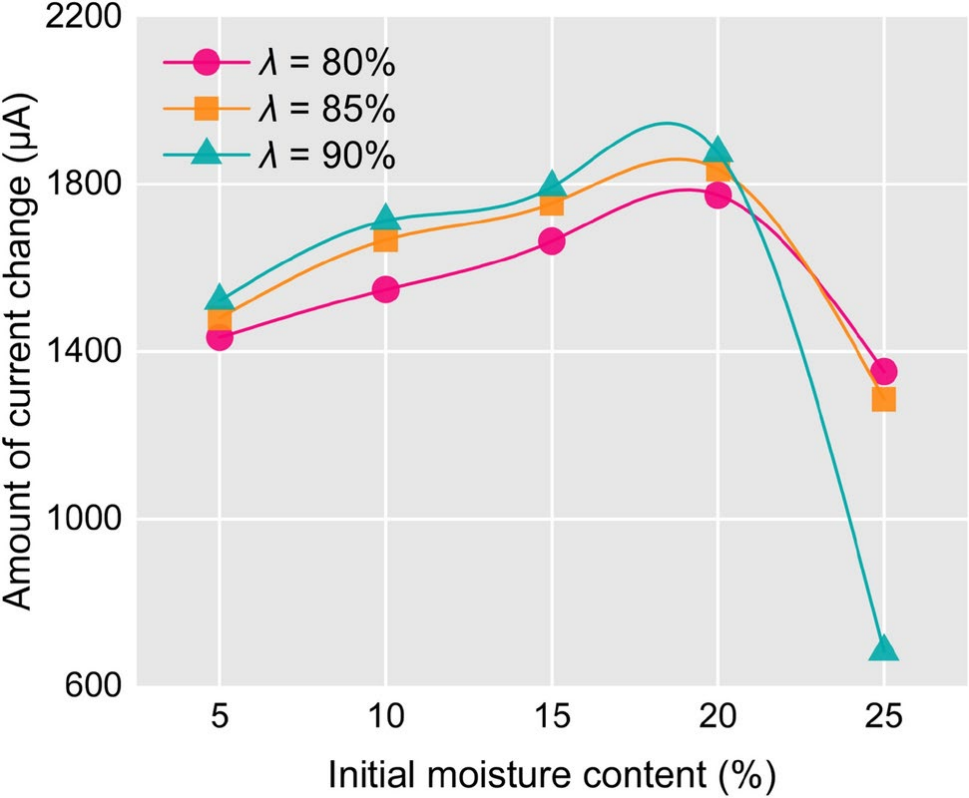
Variation of solution current at the end of disintegration.

Figure 9 reveals that when *ω* ranges from 5 to 20%, the amount of current change increases with the increase of *λ*. The reason is that when *ω* is the same, the large *λ* contains more cementitious substance. The number of ions is relatively large accordingly. When *λ* is the same, the samples with *ω* of 5–20% have the equivalent cementation substance contents. However, the amount of current change increases with the increase of *ω*. This is because the remolded soil samples are prepared by using untreated natural water. The mineral ions in natural water can augment the electric current in the solution. Samples with a high initial moisture content contain more natural water, which results in a relatively large changes of current.

When *ω* is 25%, the amount of current change is minimized. This is because the sample cannot fully disintegrate, leading to incomplete dissolution of the cementitious material. The current variation of *ω* = 25% at the end of disintegration are 1352 μA, 1286 μA, 683 μA for *λ * = 80%, 85%, 90%. This decreases trend corresponds with the relationship between maximum disintegration rate and *λ* discussed in Section "Disintegration rate and air escape rate". The aforementioned data suggest that there exists a positive correlation between the maximum disintegration rate and the final current change. Since the current variation can reveal the dissolution amount of the cementitious material; moreover, the dissolution of the cementitious substance is accompanied by the soil disintegration process. Hence, the final dissolution amount of cementitious substance is positively correlated with the maximum disintegration rate.

## Discussion

### Correlation among compaction, initial moisture content, and disintegration completion time

Section "Disintegration completion time and velocity" indicates that disintegration completion time is related to compaction degree *λ* and initial moisture content *ω*. SPSS Statistics 22.0 is utilized to explore the effect extent of *λ* and *ω* on the completion time of disintegration. A linear model (Eq. (16)) is employed to calculate the significance between compaction degree, initial moisture content, and completion time of disintegration (Table 2). This analysis aims to examine the correlation among compaction, initial moisture content, and disintegration completion time.16where *y* is the response variable; *x* is the independent variable; *b*_0_ is the intercept; *β* is the regression coefficient; *ε* is a correction constant; *i* = 1, 2, 3…*n*, *p* = 1, 2, 3…*n*.

**Table 2:** Correlation among compaction degree, initial moisture content, and disintegration completion time.

Correlation coefficient	Compaction degree (* λ*)	Initial moisture content (*ω*)
Person correlation* r*	0.204	0.930**
Significance level *P*	0.040	0.000
Number of tests	15	15

**The correlation is significant at a confidence level (two-test) of 0.01.

The Person correlation* r* is between − 1 and 1. *r* > 0 indicates a positive correlation between the variables. *r* < 0 indicates a negative correlation between the variables. *r* = 0 indicates no correlation between the variables. The large absolute *r* presents the strong correlation between variables. *P* represents the available level of significance of the coefficients in the regression formula, and the confidence interval is generally set at 95%. *P* > 0.05 indicates that the regression coefficient is not significant. When *P* ≤ 0.05, the regression coefficient is significant, indicating a significant influence between variables. A small *P* reveals the great effects between variables.

Table 2 shows that the *r* for compaction degree and initial moisture content is 0.204, 0.930, respectively. This indicates that the compaction degree and initial moisture content are both positively correlated with disintegration completion time. The *P* for compaction degree and initial moisture content is 0.040 and 0.000, respectively. Both compaction degree and initial moisture content has a significant effect on the disintegration completion time. However, the effect of initial moisture content is greater than the impact of compaction degree.

### Disintegration mechanism

**Fig. 10:**
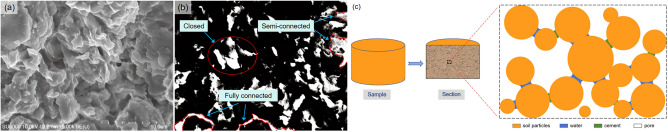
(**a**) Pore structure of GRS: scanning electron microscope image; (**b**) Corresponding binary image; (**c**) GRS skeleton model.

This section provides microcosmic perspectives on the disintegration mechanism of GRS based on Section "Test results and analysis". Unsaturated GRS consists of soil particles, water, cementitious substance, and pores. Soil skeleton is composed of quartz particles. The connection between soil particles is maintained by capillary water and cementitious substance. The pores can be classified into two types. One is fully and semi-connected to the atmosphere. The other is disconnected from the atmosphere, and refers to the closed pore space (Fig. 10a,b). A microscopic skeleton model of GRS is established based on the pore structure (Fig. 10c).

**Fig. 11:**
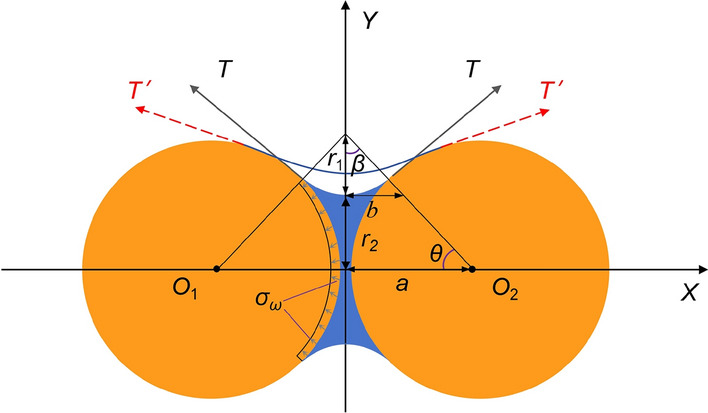
Connection model for soil–water-soil.

The inter-particle suction stress generated by capillary water (Fig. 11) and the viscous stress formed by cementation substance exerts a crucial role in maintaining soil stability. According to the formula proposed by Young-Laplace for the pressure difference at the air-water interface, the suction stress can be expressed by Eq. (17).17

where *T* is the surface tension of capillary water, in kN/m, which depends only on temperature^36^; *r*_1_ and *r*_2_ is the radii of curvature in orthogonal directions of the air–water interface, respectively. *r*_1_ and *r*_2_ can be calculated by Eqs. (18)–(20).181920

Equations (19) and (20) is substituted into Eq. (17) to obtain Eq. (21):21

**Fig. 12:**
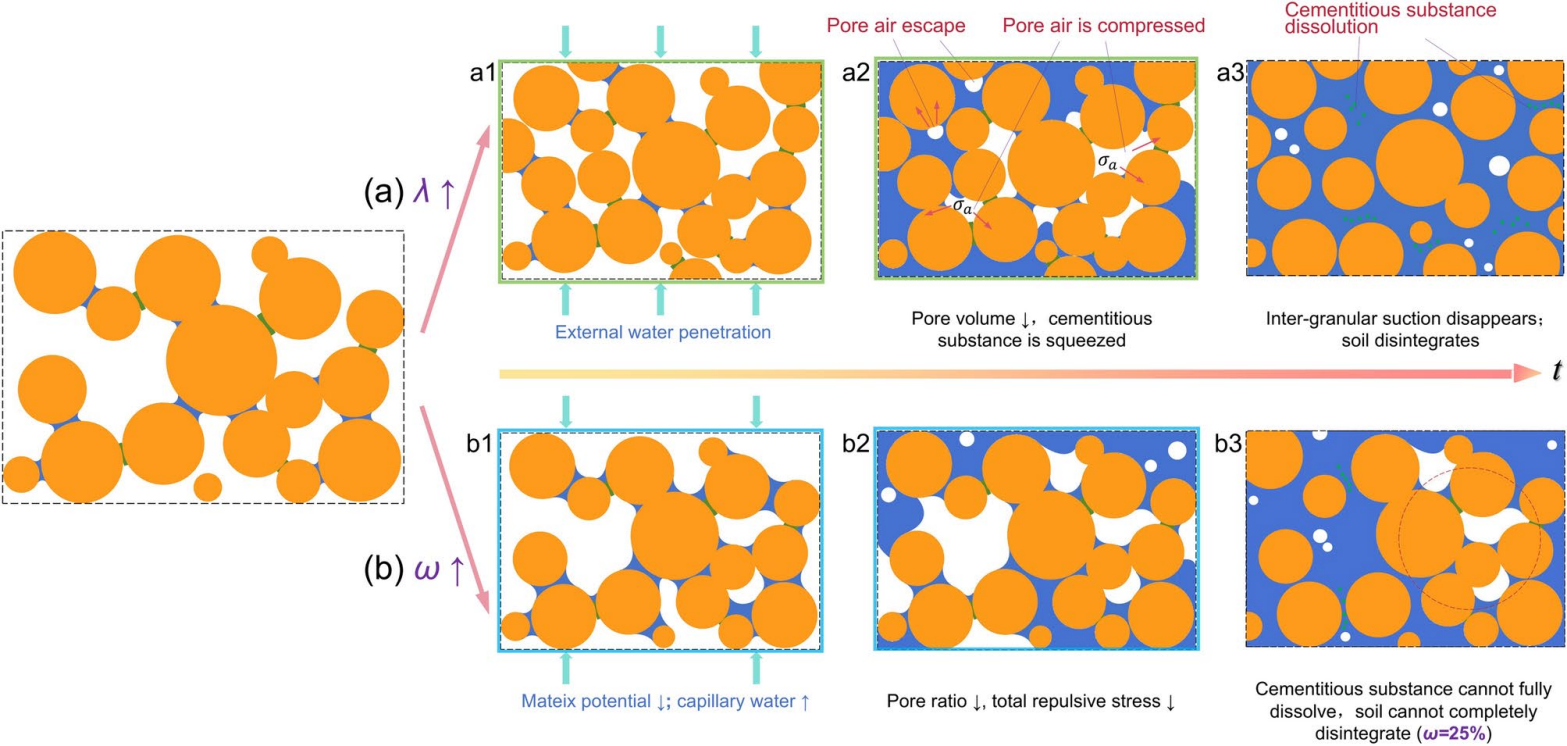
Effect of *λ* and *ω* on soil disintegration.

When the specimen is immersed in water, the presence of capillary action causes external water to continuously penetrate the soil along the pores that are fully and semi-connected to the atmosphere (Fig. 12a1). Subsequently, the air in these pores begins to escape rapidly. This process can generate disturbance to the soil structure. Concurrently, the moisture content begins to increase, leading to the rise of the capillary water level. When *a* is constant, *θ* and *b* increases with the rise of capillary water level. Equation (21) indicates that the suction stress will decrease accordingly. In addition, the cementitious substances that bond the soil particles begins to dissolve. The cohesion gradually disappears. These results lead to a continuous decrease in inter-particle suction stress between soil particles. Furthermore, when water enters the closed pores, the air inside the pores cannot be discharged and becomes compressed. This will exert compressive stress on the surrounding soil particles. The perturbing and compressive stress are collectively referred to be repulsive stress *σ*_*a*_. The suction stress of capillary water between soil particles is represented by *σ*_*ω*_. The viscous stress of the cementitious substance is represented by *σ*_*c*_. The gravitational stress of soil particles is neglected. The equilibrium relationship between these three stresses can be expressed as follows:22

If *σ*_*a*_ exceeds the total sum of *σ*_*ω*_ and *σ*_*c*_, soil particles will experience uneven stress distribution, which contributes to soil disintegration. The following analysis based on the above principle is aimed to examine the effect of compaction *λ* and initial moisture content *ω* on disintegration mechanism.

When *ω* remains constant, the increase in *λ* leads to the reduction in pore volume and the compression of cementitious substance. Consequently, the repulsive stress diminishes, while the viscous stress increases (Fig. 12a2). This is conductive to the stability of soil particles. This is the reason why the anti-disintegration property of GRS can be enhanced when *λ* increases from 80 to 90%, as described in Section "Disintegration test results". However, the viscous stress cannot be increase indefinitely, and there exists a maximum threshold. In particular, when the viscous stress reaches the threshold, it will no longer increase due to the squeezing of the cementing substance. Section "Disintegration completion time and velocity" illustrates that when *λ* is 85%, the continuous increase in *λ* has little effect on the disintegration completion time. This further validates the abovementioned observation. As water continues to infiltrate, the cementitious substance gradually dissolves, and the suction stress generated by capillary water keeps weakening. This leads to a gradual attenuation of the inter-granular suction of soil particles. Eventually, the soil experiences the disintegration as a result of the entire disappearance of inter-granular suction stress (Fig. 12a3).

When *λ* is constant, and *ω* increases from 5 to 20%, the decrease of matrix potential of soil effectively prevent smooth infiltration of external water into the soil. As a result, the cementitious substance is not prone to dissolve. In addition, the increase in capillary water further inhibits the separation of soil particles (Fig. 12b1). Furthermore, a large number of pores are occupied by capillary water. The proportion of pore air is extremely low. Most of pore air escapes in the form of tiny bubbles, which generate the small disturbing stress. The pore air that is compressed is scarce, and the corresponding stress is relatively small. This results in a relatively small total repulsive stress (Fig. 12b2). Therefore, the negative influence of total repulsive stress on soil particles is insignificant. The combined effect of these factors is the reason why the disintegration time increases with the increase of *ω*. When *ω* increases to 25%, the cementitious substance cannot fully dissolve (Fig. 12b3). The smallest variation of current change at *ω* = 25% confirms this phenomenon. The specimen with *ω* of 25% disintegrates only in the edge area, as described in Section "Disintegration test results".

## Conclusion

This study developed two test instruments to monitor the soil disintegration and the volume of escaping pore air in real-time, and observe the variation of cementation substance dissolution. The intrinsic effect mechanism of escaping pore air and cementitious substance dissolution on soil disintegration was revealed. The main conclusions are drawn as follows:

1. The initial moisture content of GRS exerts a remarkable influence on disintegration pattern, completion time and velocity. The specimens with an initial moisture content ranging from 5 to 15% undergo three stages: water absorption, rapid disintegration, and collapse disintegration. The specimens with an initial moisture content of 20% fails to present the third stage. The specimens with an initial moisture content of 25% cannot disintegrate completely. As the initial moisture content increases from 5 to 25%, the completion disintegration time is prolonged and the disintegration velocity is decelerated.
2. The effect of the increasing compaction degree on the disintegration completion time and velocity is related to the initial water content. The initial water content of 10% and 20% exhibits the minimum and maximum effects respectively. When the initial water content is 25%, the maximum disintegration velocity can decrease as compaction degree increases from 80 to 90%.
3. The entire disintegration process of GRS is accompanied by the escape of pore air and the dissolution of cementitious substances. The phenomenon of air escape in the initial stage of disintegration is rather remarkable. As the water content and compactness increase, the size of the bubbles gradually decreases. When the initial water content ranges from 5 to 20%, the final dissolution of the cementitious substances increases with the increase in compactness. However, an initial water content of 25% manifests an opposite trend; at this moment, the final dissolution of the cementitious substances is positively correlated with the maximum disintegration rate.
4. The intrinsic mechanism of GRS disintegration includes three common aspects. First, the infiltration of external water leads to an increase in soil moisture content, which reduces the suction stress generated by capillary water. Subsequently, water absorption leads to the dissolution of cementation substance, resulting in the gradual disappearance of viscous stress. Finally, the air escaping from the pores that are fully and semi-connected to the atmosphere will exert disturbances on the soil structure. In addition, the air that cannot be discharged within the closed pore space will generate compressive stress. Particularly, when the sum of compressive stress and disturbance stress exceeds the sum of suction stress and viscous stress, soil particles begin to separate from the parent body and disintegrate.

The aforementioned research results will establish a critical scientific support for us to carry out refined dynamic process simulation of GRS disintegration in the next step. Furtherly, the research and development of digital twin model of GRS disintegration for geological disasters and related geological engineering issues, as well as the intelligent control of virtual- real interactive will be gradually realized.

## Data Availability

The datasets generated during and analysed during the current study are available from the corresponding author on reasonable request.

## References

[1] Chen, W., Jian, W., Dong, Y. & Lin, X. Influence of weak structural surface on stability of granite residual soil slopes. *Chin. J. Geol. Hazard Control* **26**, 23–30. 10.16031/j.cnki.issn.1003-8035.2015.01.004 (2015).

[2] Rahman, A. S. A., Noor, M. J. M., Jais, I. B. M., Sidek, N. & Ahmad, J. in *Advances in Civil Engineering and Science Technology* Vol. 2020 (2018).

[3] Coutinho, R. Q., Silva, M. M., dos Santos, A. N. & Lacerda, W. A. Geotechnical characterization and failure mechanism of landslide in granite residual soil. *J. Geotech. Geoenviron. Eng.* **145**, 05019004. 10.1061/(ASCE)GT.1943-5606.0002052 (2019).

[4] Pham, K., Kim, D., Lee, I.-M. & Choi, H. Hydraulic-mechanical properties of unsaturated granite-weathered residual soil in Korea. *Vadose Zone J.* **18**, 1–13. 10.2136/vzj2018.10.0188 (2019).

[5] Kim, J., Jeong, S., Park, S. & Sharma, J. Influence of rainfall-induced wetting on the stability of slopes in weathered soils. *Eng. Geol.* **75**, 251–262. 10.1016/j.enggeo.2004.06.017 (2004).

[6] Zhai, Q., Rahardjo, H. & Satyanaga, A. Variability in unsaturated hydraulic properties of residual soil in Singapore. *Eng. Geol.* **209**, 21–29. 10.1016/j.enggeo.2016.04.034 (2016).

[7] Rahardjo, H., Leong, E. C. & Rezaur, R. B. Effect of antecedent rainfall on pore-water pressure distribution characteristics in residual soil slopes under tropical rainfall. *Hydrol. Process.* **22**, 506–523. 10.1002/hyp.6880 (2008).

[8] Jiao, J. J., Wang, X. S. & Nandy, S. Confined groundwater zone and slope instability in weathered igneous rocks in Hong Kong. *Eng. Geol.* **80**, 71–92. 10.1016/j.enggeo.2005.04.002 (2005).

[9] Liu, W. et al. Moisture content, pore-water pressure and wetting front in granite residual soil during collapsing erosion with varying slope angle. *Geomorphology* **362**, 107210. 10.1016/j.geomorph.2020.107210 (2020).

[10] Xia, J., Cai, C., Wei, Y. & Wu, X. Granite residual soil properties in collapsing gullies of south China: Spatial variations and effects on collapsing gully erosion. *Catena* **174**, 469–477. 10.1016/j.catena.2018.11.015 (2019).

[11] Shu, R., Kong, L., Liu, B. & Wang, J. Stress-strain strength characteristics of undisturbed granite residual soil considering different patterns of variation of mean effective stress. *Appl. Sci. -Basel* **11**, 1874. 10.3390/app11041874 (2021).

[12] Tang, L., Xu, H., Liu, Q., Sun, Y. & Wu, Y. Experimental study on disintegration characteristics of improved granite residual soil. *China J Highway Transp.* **35**, 75–87. 10.19721/j.cnki.1001-7372.2022.10.008 (2022).

[13] Rahardjo, H., Satyanaga, A., Leong, E.-C., Ng, Y. S. & Pang, H. T. C. Variability of residual soil properties. *Eng. Geol.* **141**, 124–140. 10.1016/j.enggeo.2012.05.009 (2012).

[14] Li, C., Kong, L., Shu, R., An, R. & Zhang, X. Disintegration characteristics in granite residual soil and their relationship with the collapsing gully in South China. *Open Geosci.* **12**, 1116–1126. 10.1515/geo-2020-0178 (2020).

[15] Sun, Y., Liu, Q., Xu, H., Wang, Y. & Tang, L. Influences of different modifiers on the disintegration of improved granite residual soil under wet and dry cycles. *Int. J. Min. Sci. Technol.* **32**, 831–845. 10.1016/j.ijmst.2022.05.003 (2022).

[16] Liang, S., Xiao, X., Fang, C., Feng, D. & Wang, Y. Experimental study on the mechanical properties and disintegration resistance of microbially solidified granite residual soil. *Crystals* **12**, 132. 10.3390/cryst12020132 (2022).

[17] Xia, D. et al. Effect of soil moisture on soil disintegration characteristics of different weathering profiles of collapsing gully in the hilly granitic region, South China. *Plos One* **13**, e0209427. 10.1371/journal.pone.0209427 (2018). 30596706 10.1371/journal.pone.0209427 PMC6312242

[18] Liu, X., Zhang, X., Kong, L., Wang, G. & Liu, H. Formation mechanism of collapsing gully in southern China and the relationship with granite residual soil: A geotechnical perspective. *Catena* **210**, 105890. 10.1016/j.catena.2021.105890 (2022).

[19] Saffari, P. et al. Collapse behavior of unsaturated remolded granitic residual soil. *Bull. Eng. Geol. Enviro.* **79**, 3857–3868. 10.1007/s10064-020-01789-9 (2020).

[20] Liao, L. et al. Landslide integrated characteristics and susceptibility assessment in Rongxian county of Guangxi, China. *J. Mountain Sci.* **16**, 657–676. 10.1007/s11629-017-4804-2 (2019).

[21] Hirata, Y. & Chigira, M. Landslides associated with spheroidally weathered mantle of granite porphyry induced by 2011 Typhoon Talas in the Kii Peninsula, Japan. *Eng. Geol.* **260**, 105217. 10.1016/j.enggeo.2019.105217 (2019).

[22] Lumb, P. The properties of decomposed granite. *Géotechnique* **12**, 226–243. 10.1680/geot.1962.12.3.226 (1962).

[23] Luo, X., Gao, H., He, P. & Liu, W. Experimental investigation of dry density, initial moisture content, and temperature for granite residual soil disintegration. *Arab. J. Geosci.* **14**, 1060. 10.1007/s12517-021-07239-4 (2021).

[24] Li, C., An, R., Shu, R. & Kong, L. Initial-disintegration analysis of granite residual soil and approximate simulation of mathematical morphology. *Chin. J. Rock Mech. Eng.* **39**, 845–854. 10.13722/j.cnki.jrme.2019.0704 (2020).

[25] Chen, Y. et al. Disintegration characteristics of remolded granite residual soil with different moisture contents. *Sustainability* **16**, 84. 10.3390/su16010084 (2024).

[26] Liu, W., Song, X., Huang, F. & Hu, L. Experimental study on the disintegration of granite residual soil under the combined influence of wetting-drying cycles and acid rain. *Geomatics Nat. Hazareds Risk* **10**, 1912–1927. 10.1080/19475705.2019.1651407 (2019).

[27] Liu, X., Zhang, X., Kong, L., Wang, G. & Lu, J. Disintegration of granite residual soils with varying degrees of weathering. *Eng. Geol.* **305**, 106723. 10.1016/j.enggeo.2022.106723 (2022).

[28] Zhou, X., Liu, P. & Lan, Z. Experimental study on disintegration behavior of granite residual soil. In *Advances in Transportation Geotechnics and Materials for Sustainable Infrastructure*, 98–104. 10.1061/9780784478509.013 (2014).

[29] Rahardjo, H., Aung, K. K., Leong, E. C. & Rezaur, R. B. Characteristics of residual soils in Singapore as formed by weathering. *Eng. Geol.* **73**, 157–169. 10.1016/j.enggeo.2004.01.002 (2004).

[30] Sun, Y. & Tang, L. Use of X-ray computed tomography to study structures and particle contacts of granite residual soil. *J. Central South Univ.* **26**, 938–954. 10.1007/s11771-019-4062-2 (2019).

[31] Tang, L., Chen, Y., Zhou, Z. & Cheng, Z. Study on the influence of grain size composition on engineering properties of granite residual soil. *Polish J. Environ. Stud.* **32**, 4291–4300. 10.15244/pjoes/166593 (2023).

[32] Liu, W., Song, X., Luo, J. & Hu, L. The processes and mechanisms of collapsing erosion for granite residual soil in southern China. *J. Soils Sediments* **20**, 992–1002. 10.1007/s11368-019-02467-4 (2020).

[33] Zhang, X., Liu, X., Chen, C., Xu, Y. & Liu, H. Evolution of disintegration properties of granite residual soil with microstructure alteration due to wetting and drying cycles. *Bull. Eng. Geol. Environ.* **81**, 93. 10.1007/s10064-022-02602-5 (2022).

[34] Ze, Z., Vadim, P., Svetlana, N., Zhang, Z. & Wu, J. Disintegration characteristics of a cryolithogenic clay loam with different water content: Moscow covering loam (prQIII), case study. *Eng. Geol.* **258**, 105159. 10.1016/j.enggeo.2019.105159 (2019).

[35] Li, X.-A., Wang, L., Yan, Y.-L., Hong, B. & Li, L.-C. Experimental study on the disintegration of loess in the Loess Plateau of China. *Bull. Eng. Geol. Environ.* **78**, 4907–4918. 10.1007/s10064-018-01434-6 (2019).

[36] Zhang, S. & Tang, H. Experimental study of disintegration mechanism for unsaturated granite residual soil. *Rock Soil Mech.* **34**, 1668–1674. 10.16285/j.rsm.2013.06.009 (2013).

[37] Wang, J., Xiang, W. & Bi, R. Experimental study of influence of matric suction on disintegration of unsaturated remolded loess. *Rock Soil Mech.* **32**, 3258–3262. 10.3969/j.issn.1000-7598.2011.11.010 (2011).

[38] Qi, Y. et al. Influences of soil disintegration in water on slope stability. *Chin. J. Geotech. Eng.* **42**, 214–218. 10.11779/CJGE2020S2038 (2020).

[39] Gu, T., Yuan, L., Hu, W., Zhu, L. & Wang, X. Experimental research on disintegration of the Heifangtai loess. *Hydrogeol. Eng. Geol.* **44**, 62–70. 10.16030/j.cnki.issn.1000-3665.2017.04.10 (2017).

[40] Loginov, P. V., Salikhova, Z. R. & Sultanov, K. S. Experimental and theoretical method for determining mechanical characteristics of soils under dynamic loads. *Mech. Solids* **54**, 915–928. 10.3103/S0025654419060074 (2019).

[41] Adedokun, S. I. et al. Geotechnical beneficiation of the strength indices of lateritic soil using steel slag and cement. *Int. J. Eng. Res. Africa* **59**, 101–117. 10.4028/p-e13k1f (2022).

[42] Zhou, C., Jing, X. & Liu, Z. Disintegration characteristics haracteristics and modification of weathered soil in red beds in southern China. *J. Eng. Geol.* **27**, 1253–1261. 10.13544/j.cnki.jeg.2018-288 (2019).

[43] Große, A.-K., Cantré, S. & Saathoff, F. The applicability of disintegration tests for cohesive organic soils. *J. Environ. Eng. Landscape Manag.* **23**, 1–14. 10.3846/16486897.2014.919924 (2015).

[44] Wang, N., Wang, Q., Xue, Q. & Liu, X. Experimental study of static disintegration on unsaturated soil. *Appl. Mech. Mater.* **580–583**, 68–72. 10.4028/www.scientific.net/AMM.580-583.68 (2014).

[45] Zhu, L., Fan, H. & Ma, R. Effects of freeze-thaw cycles and soil water contents on disintegration characteristics of brown earth. *Acta Pedologica Sinica* **60**, 77–88. 10.1111/sum.12967 (2023).

[46] Wang, J., Gu, T., Zhang, M., Xu, Y. & Kong, J. Experimental study of loess disintegration characteristics. *Earth Surf. Process. Landforms* **44**, 1317–1329. 10.1002/esp.4575 (2019).

[47] Guhra, T., Ritschel, T. & Totsche, K. U. Formation of mineral-mineral and organo-mineral composite building units from microaggregate-forming materials including microbially produced extracellular polymeric substances. *Eur. J. Soil Sci.* **70**, 604–615. 10.1111/ejss.12774 (2019).

[48] Tan, W., Xu, Y., Shi, Z., Cai, P. & Huang, Q. The formation process and stabilization mechanism of soil aggregates driven by binding materials. *Acta Pedologica Sinica* **60**, 1297–1308. 10.11766/trxb202308060312 (2023).

